# Validity and reliability of the Persian version of the oropharyngeal Mucositis quality of life scale

**DOI:** 10.1186/s12903-021-01938-w

**Published:** 2021-11-23

**Authors:** Fatemeh Sadat Hasheminasab, Mona Pourpasha, Azizallah Dehghan, Mahboubeh Yari Galousalari, Seyed Mehdi Hashemi, Mohammad Setayesh

**Affiliations:** 1 Pharmacology Research Center, Zahedan University of Medical Sciences, Zahedan, Iran; 2 Department of Oral and Maxillofacial Medicine, Dentistry School, Kashan University of Medical Sciences, Kashan, Iran; 3 Noncommunicable Diseases Research Center, Fasa University of Medical Sciences, Fasa, Iran; 4 Department of Hemato-Oncology, Bahonar Hospital, Kerman University of Medical Sciences, Kerman, Iran; 5 Clinical Immunology Research Center, Ali-Ebne Abitaleb Hospital, Zahedan University of Medical Sciences, Zahedan, Iran; 6 Department of Traditional Medicine, School of Persian Medicine, Kerman University of Medical Sciences, Kerman, Iran

**Keywords:** Oral mucositis, Validity, Reliability, OMQoL, Quality of life, Iran

## Abstract

**Background:**

Oral mucositis is one of the serious complications of chemotherapy and/or radiotherapy that significantly affects the quality of patients’ life. The Oropharyngeal Mucositis-Specific Quality-of-Life questionnaire (OMQoL) is an acceptable instrument for measuring the quality of life in these patients. The aim of this study was to evaluate the validity and reliability of the Persian version of OMQoL questionnaire.

**Methods:**

This study was a cross-sectional and multi-centric research. After translation from English to Persian, back translation, and cultural adaptation, the Persian version of the questionnaire was prepared. One hundred forty-four patients suffering from oral mucositis referred to three different university affiliated hospitals related to Zahedan, Kerman and Tehran Universities of Medical Sciences, were participated in this study. Then the questionnaire was completed by trained interviewers. The reliability was assessed by Cronbach’s alpha coefficient, and validity was measured through factor analysis, and construct validity (including discriminant validity, and convergent validity) methods.

**Results:**

Cronbach's alpha in all dimensions was higher than 0.9 that indicated a perfect internal consistency. The results of factor analysis indicated that the dimensions specified in the Persian version were the same as the original version (Factor loading of all items > 0.4). The correlation coefficient for all items was more than 0.75, and the convergence validity was 100% in all dimensions.

**Conclusion:**

The results of this study showed that the reliability, and validity of Persian version of OMQoL were acceptable, which can be used to measure the quality of life in patients with oral mucositis.

## Introduction

Oral mucositis is a complication of the cancer treatment with mucotoxic agents that appear with inflammation and/or ulcer of the oral mucosa. The incidence of mucositis has a wide range from 5 to 40% in the patients with solid tumors undergoing chemotherapy to nearly 100% of patients receiving radiotherapy for head, and neck cancer [1, 2]. These lesions may occur 3 to 10 days after initiation of cancer therapy, and involve, the floor of the mouth, labial and buccal mucosa, the tongue, and soft palate [2, 3]. Oral mucositis can adversely affect patients’ health status, and reduce their quality of life [4]. It can cause painful lesions, secondary infections, and consequently sepsis, difficulty in chewing, drinking, and swallowing, and then decreasing nutritional uptake [5, 6]. Patients with oral mucositis, especially those subjected to hematopoietic stem cell transplantation experience distressing conditions. Inadequate consumption of fluid, and foods may aggravate the clinical status and can lead to periods of dehydration and malnutrition. This condition threatens not only physical but also mental health. It has been demonstrated that about 38% of patients with oral mucositis suffer from depression [5]. Economic consequences and medicinal resources usage for management of this disease are considerable. A study showed that treatment of chemo-radiotherapy induced oral mucositis due to lung and head, and neck cancer possess over 17,000$ (USD) incremental cost per patient [7]. So for the evaluation outcome of patients with oral mucositis to reflect the full impact of oral condition, assessment of the quality of life as a critical health-related indicator, besides the biomedical parameters is necessary [8, 9]. The definition of quality of life, according to the World Health Organization is “an individual’s perception of their position in life in the context of the culture, and value systems in which they live, and in relation to their goals, expectations, standards, and concerns” [10].

It is mostly considered that health-related quality of life is a subjective and multi-dimensional construct encompassing many aspects such as physical symptoms, emotional functioning, physical functioning, role functioning and social functioning and well-being [11, 12]. Because many dimensions have the potential to contemplate, there is a controversy among researchers to which aspects should be included for evaluating health-related quality of life [12].

The Oropharyngeal Mucositis-specific Quality-of-Life (OMQoL) tool including 31 items categorized into four dimensions that evaluate the quality of life in patients with oral mucositis. This instrument covers most of the aspects that are important in oral mucositis, including symptoms, nutrition, social function, and swallowing symptoms [8].

Iran has about 80 million population speaking Farsi (Persian) except for the Tajikistan and Afghani people. The necessity of applying valid and reliable tools for assessment of the quality of life-related to oral mucositis, on the other hand, the large population who speak Persian in the world, demonstrate the importance of the standardization an appropriate questionnaire particularly evaluates the quality of life in patients with oral mucositis in Persian. So the goal of this study was to develop the Persian version of the OMQoL to assessing quality of life in patients with oral mucositis due to mucotoxic cancer agents.

## Materials and methods

### Translation and cultural adaptation

The protocol for this study was approved by the ethics committee of Zahedan University of Medical Sciences (IR.ZAUMS.REC.1400.177). The translation and cultural adaptation were processed in six specific steps, according to internationally standardized and accepted guidelines [13, 14].

In the “forward translation” step, the English version of the OMQoL questionnaire was independently translated into Persian by an expert team including three linguists and a physician (whose mother tongues are Persian and who are expert in English); they noted all linguistic and cultural concepts to discuss and resolve any disagreement in a team meeting. Next, the second step was taken and "synthesis of the translations" was performed. The expert committee reviewed and discussed all four translations of previous stage; after consensus, a single agreed Persian version was obtained. At the next step, the Persian version was back-translated by a different linguists’ team (two professional translators who were blind to the original version). In this phase, the expert committee evaluated whether Persian OMQoL version linguistically represent the content of the original version.

The cultural adaptation, and conceptual compatibility of the Persian version were evaluated in the fourth step. Also, the attempt was to detecting linguistic and interpretation mistakes in order to create final Persian version for the pilot study. The fifth step was conducting a pilot study for pre-test the Persian version questionnaire. Based on the pilot study, all items were comprehensible; therefore, no changes were necessary, and thus the final Persian version was prepared.

### Sample size estimation

Based on Fayer et al*.*’s study, the appropriate sample size for assessment reliability and validity of quality of life questionnaires is 100 to 400 people [15]. A total of 144 patients with oral mucositis were included in this study. Kaiser–Meyer–Olkin (KMO) index was calculated to measure the sample adequacy factor analysis.

### Participants

This study was conducted in the three regional university-affiliated hospitals in Iran. From November 2018 to March 2019, 144 patients with oral mucositis referred to hemato-oncology ward of Bahonar hospital, outpatient oncology clinic of Khatam hospital, and cancer institute of Imam Khomeini hospital respectively affiliated to Kerman University of Medical Sciences, Zahedan University of Medical Sciences and Tehran University of Medical Sciences were included in this study. The informed consent was received from all patients. Patients were examined by specialists using the WHO oral mucositis scale for determining the grade of mucositis. This scale is as follows:

- Grade 0: no mucositis
- Grade 1: Soreness with or without erythema, No ulceration
- Grade 2: erythema, ulcers, swallowing solid diet is possible
- Grade 3: ulcers, erythema, swallowing only liquid diet is possible
- Grade 4: ulcers, alimentation is impossible.

Patients completed the questionnaire, and in the cases of illiteracy, the questionnaire was filled by interview.

### Instrument

The OMQoL questionnaire exclusively evaluates the quality of patients’ life with oral mucositis. This instrument consists of 31 items, categorized into four dimensions that assess symptomatology (9 items), nutrition (10 items), social function (7 items), and swallowing status (5 items) using a 4 point Likert scale (not at all, a little bit, quite a bit, very much respectively from 1 to 4 score). A higher score indicates a lower quality of life.

### Validity, reliability and statistical analysis

Cronbach’s alpha coefficient was applied for evaluating the internal consistency to determine reliability. The values of Cronbach’s alpha’s range is between zero and one, and the closer to one, the greater its reliability [16].

Identification of the questionnaire dimensions and comparison with the original version of the instrument were done by using factor analysis. Factor loading above 0.4 was considered significant [17]. Exploratory factor analysis is a multivariate analysis method used to identify the dimensions of a questionnaire. This method shows whether the dimensions measured by the translated questionnaire are similar to the original version or not. In this study, the Varimax rotation was used to better identify the factors [18].

Spearman’s correlation coefficient was used for assessing construct validity including convergent validity (correlation coefficient between each item and the total score of the respective dimension) and discriminant validity (correlation coefficient between each item and the total score of the other dimensions). In convergence validity, all one-dimensional items must have a relatively strong correlation with its own dimension. Values equal to or greater than 0.75 indicate strong correlation [19]. In discriminant validity, the correlation coefficient of an item with other dimensions of the questionnaire should be less than its own dimension. A correlation of less than 0.4 with other dimensions is excellent for any item [18].

Statistical analysis was carried out by SPSS 0.22.

## Results

### Demographic information

The mean age of the participants was 53.9 ± 14.7 years (range, 18–79 years). Most of the patients were diagnosed with nasopharynx cancer, and half of them received chemo-radiotherapy. Table 1 shows other demographic and clinical characteristics of the patients.

**Table 1:** Demographic and Characteristics data of Study Participants (N = 144)

Age (years), mean (SD)	53.9 (14.7)
*Sex,* n (%)	
Men	64 (44.4)
Women	80 (55.6)
*Educational level,* n (%)	
illiterate	10 (6.9)
Elementary	31 (21.5)
High school or diploma	66 (45.8)
Academic education	37 (25.7)
*Cancer diagnosis,* n (%)	
nasopharynx	98 (68.1)
breast	10 (6.9)
ALL	6 (4.2)
AML	5 (3.5)
other	25 (17.4)
*Cancer therapy,* n (%)	
Chemotherapy	31 (21.5)
Radiotherapy	41 (28.5)
Chemo-radiotherapy	72 (50)
*WHO grade for mucositis,* n (%)	
1	16 (11.1)
2	34 (23.6)
3	48 (33.3)
4	46 (31.9)

### Reliability

Table 2 presents mean ± SD and the value of Cronbach’s alpha coefficients for different dimensions. The patients had the most problem in the diet dimension (highest score) and the lowest in the swallowing dimension (lowest score). Cronbach’s alpha coefficients were higher than 0.9 for all dimensions indicating internal consistency and appropriate reliability of the instrument.

**Table 2:** Mean and Cronbach’s alpha Coefficients for each dimension of OMQoL questionnaire (N = 144)

Dimensions	Mean (SD)	Cronbach’s alpha (total)	Cronbach’s alpha (men)	Cronbach’s alpha (women)
Symptoms	27.72 (7.9)	0.97	0.97	0.96
Diet	32.21 (7.7)	0.95	0.95	0.95
Social function	20.12 (7.2)	0.97	0.98	0.96
Swallowing	14.54 (5.3)	0.97	0.97	0.96

### Validity

Table 3 presents the results of factor analysis with the Varimax rotation. Factor loading of all items was above 0.4. These results indicate that the dimensions specified in the Persian version are the same as the original version, and the items in each dimension are in their respective dimensions. The Kaiser–Meyer–Olkin (KMO) index was 0.741, which indicates the adequacy of the sample size.

**Table 3:** Result of factor analysis

Items	Symptoms	Diet	Social function	Swallowing
Item 1	0.845			
Item 2	0.786			
Item 3	0.792			
Item 4	0.845			
Item 5	0.859			
Item 6	0.755			
Item 7	0.808			
Item 8	0.778			
Item 9	0.867			
Item 10		0.777		
Item 11		0.875		
Item 12		0.856		
Item 13		0.684		
Item 14		0.791		
Item 15		0.775		
Item 16		0.743		
Item 17		0.820		
Item 18		0.847		
Item 19		0.827		
Item 20			0.769	
Item 21			0.821	
Item 22			0.915	
Item 23			0.831	
Item 24			0.921	
Item 25			0.907	
Item 26			0.884	
Item 27				0.861
Item 28				0.896
Item 29				0.896
Item 30				0.904
Item 31				0.911

Table 4 shows the results of item-convergent and item-discriminant validity. The correlation coefficients for all items were more than 0.75, and the convergence validity was 100% in all dimensions.

**Table 4:** Item Scaling Tests: Convergent and Disconvergent validity for OMQoL dimensions

Dimensions	Number of Items	Item-Convergent Validity (Range of Correlation)	Correlation > 0.4	Scaling success	Item-Discriminant Validity (Range of Correlation)
Symptoms	9	− 0.94	9	100%	0.15–0.43
Diet	10	0.78–0.90	10	100%	0.37–0.50
Social function	7	0.90–0.96	7	100%	0.18–0.26
Swallowing	5	0.92–0.95	5	100%	0.23–0.34

## Discussion

According to recent studies, there are several tools that have been utilized to measure the quality of life in patients with oral mucositis. Functional Assessment of Cancer Therapy-General Scale (FACT-G) is one of these instruments, this tool has not enough sensitivity and it cannot differentiate oral mucositis in the patients group [8, 20]. Another generic health-related quality of life questionnaire is the European Organization for Research and Treatment of Cancer Quality of Life Questionnaire Core 30 (EORTC QLQ-C30). Neither FACT-G nor EORTC QLQ-C30 cover dimensions, which are specifically relevant to oral mucositis [20, 21]. In designing instruments such as the EORTC Quality of Life-Head and Neck 35 Questionnaire (EORTC QLQ-H&N35), the Performance Status Scale for Head and Neck Cancer (PSS-HN), and the FACT-Head and Neck Scale (FACT-H&N), oral dysfunction such as speech and swallowing have been considerate [8, 22, 23]. They mainly focus on some persistent disorders of salivary glands, connective tissue, vasculature, muscle, and bone because of head and neck surgery or radiotherapy, but they are unable to express problems, which are distinctly due to acute erythema and ulceration of oral mucosa in radiotherapy and chemotherapy [8]. Oral Health Impact Profile-14 (OHIP-14) is another instrument. Although, it is wildly applied in clinical trials on oral mucositis, this tool does not cover dimensions, which describe symptoms and signs usually reported by patients with oral mucositis [24].

Some other instruments such as Patient-Reported Oral Mucositis Symptoms (PROMS) scale, evaluates symptoms of oral mucositis affecting the quality of life. This scale includes a 100 mm visual analog scale that assesses ten items i.e., mouth pain, difficulty speaking, restriction of speech and, etc. [25]. The Oral Mucositis Weekly Questionnaire-Head and Neck Cancer (OMWQ-HN) is another instrument that measures the impact of oral mucositis on symptoms such as mouth and throat pain and also its effects on well-being and function [26].

OMQol instrument was developed and preliminary validated to measure oral mucositis specific health-related quality of life. This tool assesses the quality of life in patients with oral mucositis from the patient’s view. OMQol, including 31 items categorized into four dimensions and cover most of the aspects that are important in oral mucositis, including symptoms, nutrition, social function, and swallowing symptoms [8].

In previous study, the OMQoL was analyzed based on the Rasch model with a multicenter approach. According to this analyzing, the four dimensions of OMQoL indicated the same category thresholds, and the indices and reliability of the person separation were high, demonstrating their acceptable discriminatory capacity. However, there are some items in this instrument which may not be effectively targeted to the low grade oral mucositis patients [27].

For the transcultural adaptation of the Brazilian version of the OMQoL questionnaire, a study was conducted and specific steps were followed including translation, back translation, testing pre-final version on 40 participants, and completion final version. Two questions were changed to the final version due to doubts generation. At last the OMQoL Brazilian version was translated and adapted. It reflected the original tool in an adequate manner [28].

Recently, the validity and reliability of the Spanish version of the OMQoL questionnaire were assessed and 193 pediatric patients aged 8–16 years participated. In this multi-centric cross-sectional study, after translation and adaptation, reliability was determined by Cronbach's alpha and validity was measured using exploratory factor analysis and construct validity. The load of factorial analysis was more than 0.4 in four dimensions, and Cronbach's alpha was calculated 0.954. According to this study, OMQoL -Spanish is a valid and reliable tool for measuring the quality of life in children suffering from oral mucositis [29].

The current study was conducted to assess the validity and reliability of the Persian version of the OMQoL questionnaire in Iran. Cronbach's alpha in all dimensions was higher than 0.9 indicating a perfect internal consistency. In this research, the validity of OMQoL questionnaire was measured and scaling success in all dimensions was 100%.

## Conclusion

This study demonstrated that the validity and reliability of the Persian version of the OMQoL questionnaire are appropriate enough for measuring the quality of life in patients with oral mucositis. The Persian version of OMQoL is a suitable tool for evaluating the various aspects of the quality of life in patients suffering from oral mucositis both in practice and clinical research.

## Data Availability

Not applicable.

## Abbreviations

OMQoL: Oropharyngeal Mucositis-Specific Quality-of-Life questionnaire
FACT-G: Functional Assessment of Cancer Therapy-General Scale
EORTC QLQ-C30: European Organization for Research and Treatment of Cancer Quality of Life Questionnaire Core 30
EORTC QLQ-H&N35: EORTC Quality of Life-Head and Neck 35 Questionnaire
PSS-HN: Performance Status Scale for Head and Neck Cancer
FACT-H&N: FACT-Head and Neck Scale
OHIP-14: Oral Health Impact Profile-14
PROMS: Patient-Reported Oral Mucositis Symptoms
OMWQ-HN: Oral Mucositis Weekly Questionnaire-Head and Neck Cancer
